# A Case of Worms in the Urinary Bladder

**Published:** 1875-06

**Authors:** 


### A Case of Worms in the Urinary Bladder.

Dr. Melvin Rhorer reports (*Am. Practitioner*, March, 1875), the following case which he was called to see in consultation : The patient, aged sixty-four years, was a farmer, who had for the past twelve months been affected with occasional interruptions to the flow of urine, which for the last three weeks had increased in severity, causing great pain in evacuating the bladder, and which now amounted to almost a total retention. His bladder was very much distended, he having passed no urine for forty-eight hours, except a constant dribbling of highly-colored urine, with an occasional drop or two of blood.

Dr. R. easily introduced a catheter and evacuated the bladder, finding in the vessel forty or fifty small red worms about half an inch in length, and having a number of legs arranged in two distinct rows from one extremity to another, and their bodies being encircled with numberless small cartilaginous rings. It was with some difficuity that he pressed a lancet through the body. In about two hours the patient, at his suggestion, forced a passage from his bladder, amounting to several ounces of urine, with about half a dozen more worms. No attempt was made to sound for stone, the diagnosis being too clear as to the cause of the trouble. We ordered spirits of turpentine internally, and the catheter to be employed daily. For the following ten days he passed from four to six worms every action ; since which time he has voided urine without the use of the instrument, and the discharge of worms had ceased. He has no pain on micturition, and is free from his late trouble, except a sense of soreness over the hypogastric region.

[For references to similar cases the reader is referred to Gross *On the Urinary Organs*, and Roberts *On Urinary and Renal Diseases*, 2d ed., Phila , 1872, p. 590].—*Monthly Abstract Medical Sciences.*

